# Dietary patterns are associated with blood lipids at 18-year-olds: a cross-sectional analysis nested in the 1993 Pelotas (Brazil) birth cohort

**DOI:** 10.1186/s12937-018-0389-z

**Published:** 2018-08-22

**Authors:** Juliana dos Santos Vaz, Romina Buffarini, Gilberto Kac, Renata Moraes Bielemann, Isabel Oliveira, Ana Baptista Menezes, Maria Cecilia Formoso Assunção

**Affiliations:** 1 Faculty of Nutrition, Federal University of Pelotas, Rua Gomes Carneiro, 1, 2° andar, Pelotas, RS 96010-610 Brazil; 2 Epidemiology, Federal University of Pelotas, Rua Marechal Deodoro, 1160, 3° andar, Pelotas, RS 96020-220 Brazil; 3 Institute of Nutrition, Federal University of Rio de Janeiro, Avenida Carlos Chagas Filho, 367, CCS – Bloco J – 2° andar, sala 29, Cidade Universitária – Ilha do Fundão, Rio de Janeiro, RJ 21941-590 Brazil; 4 Institute of Biology, Federal University of Pelotas, Campus Capão do Leão, s/n, Capão do Leão, RS 96010-900 Brazil

**Keywords:** Dietary patterns, Food consumption, Adolescents, Lipoproteins, Cross-sectional studies

## Abstract

**Background:**

Evidence regarding the deleterious effects of diet on blood lipids in adolescence has been inconsistent, and few studies have investigated this association using a dietary pattern approach. We examined whether dietary pattern of adolescents are associated with blood lipid concentrations.

**Methods:**

Cross-sectional analysis of 3524 18-year-old participants in the 1993 Pelotas (Brazil) Birth Cohort Study. A semi-quantitative food frequency questionnaire was administered. Dietary patterns were established using principal component analysis and analysed as tertiles of factor scores. Independent associations between each dietary pattern tertile and blood lipid values (total cholesterol, LDL-cholesterol, HDL-cholesterol and triglycerides) were tested using adjusted linear regression models stratified by sex. Triglycerides were log-transformed due to their skewed distribution, and the beta coefficients should be interpreted as the % change (increase or decrease).

**Results:**

Four dietary patterns were derived: Meat Products and Fast Foods; Fruits and Vegetables; Candies, Sodas and Dairy Products; and Common Brazilian Foods. In the adjusted models, which compared the highest and lowest tertiles of dietary pattern scores, we observed that among girls: 1) the third tertile of the Meat Products and Fast Foods pattern was associated with 1.5 mg/dL (95% CI -3.05;  –0.04) lower HDL-cholesterol; 2) the second and third tertile of the Candies, Sodas and Dairy Products pattern was associated with 5% and 10% higher triglycerides (β 1.05, 95% CI 1.01; 1.09, β 1.10, 95% CI 1.05; 1.16), respectively; 3) the second and third tertiles of the Common Brazilian Foods pattern were associated with 4 mg/dL (β − 4.30, 95% CI -7.75;  –0.85, β − 4.95, 95% CI -8.53;  –1.36, respectively) lower total cholesterol and 6% lower triglycerides (β 0.94, 95% CI 0.90; 0.99, β 0.93, 95% CI 0.89; 0.98, respectively). For boys, 4) the third tertile of the Common Brazilian Foods was associated with 4.6 mg/dL (95% CI -7.91;  –1.37) lower total cholesterol and 3.8 mg/dL (95% CI -6.51; − 1.13) lower LDL-cholesterol.

**Conclusions:**

Dietary patterns were more closely associated with blood lipids among girls than boys at age 18. Higher scores for the Common Brazilian Foods pattern were associated with lower total cholesterol in both sexes.

**Electronic supplementary material:**

The online version of this article (10.1186/s12937-018-0389-z) contains supplementary material, which is available to authorized users.

## Background

The measurement and surveillance of blood lipids have become standard medical and public health practices for the early detection of cardiovascular disease risk [1, 2]. Blood lipid profiles in childhood are strongly related to adult values [3, 4]. The occurrence of dyslipidaemia in youth populations highlights the need to understand the influencing factors and the need to initiate prevention measures [1, 5]. Serum cholesterol fractions and triglycerides are determined by genetics and lifestyle factors [6–8], including eating habits [9]. A variety of dietary components, e.g., the solubility of dietary fibre, quality of carbohydrate, types of fats and antioxidant consumption, affect serum lipoproteins and triglycerides [9]. All these dietary components are determined by the source and the degree of industrial food processing of cereals, fruits and vegetables, dairy products, meats, and oils, e.g. the degree of refined grains and cereals affect the content of fibre and the availability of the carbohydrate [10].

Brazil has recently issued a new dietary guidelines focused on traditional eating habits to promote the consumption of traditional or minimally processed foods (e.g. fruits, seeds, leaves, roots, meats, eggs, milk, and any other natural foods altered by minimal processes such as removal of inedible parts, drying, boiling, pasteurisation). This is in lieu of processed (e.g. products made by adding sugar, oil, salt to increase durability of natural foods) and ultra-processed ones (e.g. snacks, drinks, ready to eat meals and many other products which often contain flavours, colours and other additives that imitate or intensify the sensory qualities) [11]. The guideline considers evidences of the increased ultra-processed food availability and intake [12] with obesity [13]. The most prevalent lipid alterations reported in a recent school-based study of Brazilian adolescents were low HDL-cholesterol and hypercholesterolaemia [5]. Studies of adolescents have shown that high saturated fat, sodium, and sugar consumption and low fruit and vegetable consumption is associated with dyslipidaemia [14, 15].

Dietary pattern analysis has emerged as a practical way to deliver information to the public and to implement policies in contrast to investigations of dietary risk factors focused on food groups or nutrients [16]. Evidence regarding the deleterious effects of diet on blood lipids in adolescence has been inconsistent [9, 17], and few studies have investigated this association using a dietary pattern approach [18, 19].

Thus, the present study sought to establish the dietary patterns of 18-year-old adolescents and to examine the possible associations of these patterns with blood lipids concentrations (total cholesterol, LDL-cholesterol, HDL-cholesterol and triglycerides) using data from the 1993 Pelotas Birth Cohort Study birth cohort study conducted in Pelotas, a city in southern Brazil. We hypothesized that higher scores on a dietary pattern characterized by traditional or minimally processed foods would be associated with a better lipid profile (i.e. lower total and LDL-cholesterol, triglycerides, and higher HDL-cholesterol), and higher scores on a pattern characterized by sweets and processed/ultraprocessed foods would be associated with a worse lipid profile (i.e. higher triglycerides, total and LDL-cholesterol and lower HDL-cholesterol).

## Methods

### 1993 Pelotas birth cohort

**Fig. 1:**
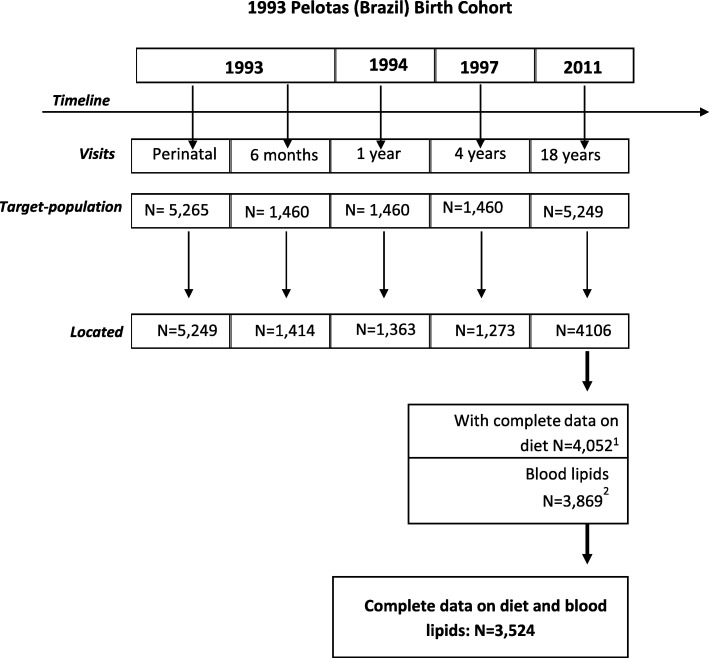
Description of the 1993 Pelotas (Brazil) Birth Cohort. ^1^Subjects excluded of diet data: did not complete the food frequency questionnaire (*n* = 22) and implausible energy report (*n* = 205). ^2^Subjects excluded regarding blood lipids: refused to provide blood samples (*n* = 180), pregnancy (*n* = 57) and breastfeeding (*n* = 92), reported use of medication for glucose or lipid control [insulin (*n* = 9), metformin (*n* = 2), statins (*n* = 2)], had haemoglobin A1c ≥6.5% (*n* = 12), one case of hypertriglyceridaemia (triglycerides> 8000)

The present study uses data from the 1993 Pelotas Birth Cohort, a prospective population-based study carried out with all newborns in Pelotas. This cohort includes all hospital births to mothers living in the urban area of the city from January 1 to December 31, 1993 (*n* = 5265) [20]. The initial cohort comprised 5249 eligible newborns (the mothers of 16 newborns declined to participate). Periodic follow-ups were conducted beginning at 3 months of age to assess several socioeconomic, health and nutritional features. The 18-year follow-up visit started in September 2011 and finished in April 2012. Of the original cohort of 5249 subjects, 4106 were interviewed and 979 were considered losses. Those who completed the interviews, added to those known to have died (*n* = 164), represented a retention rate of 81.3%. At a visit to the research clinic unit, participants provided informed consent and underwent a series of assessments that consisted of questionnaires, anthropometric measures, and blood sample collection. Those who refused to provide blood samples (*n* = 180) or reported being pregnant (*n* = 57) were excluded from blood collection, reported the use of medication for glucose or lipid control [insulin (*n* = 9), metformin (*n* = 2), statins (*n* = 2)], had haemoglobin A1c ≥6.5% (*n* = 12), were breastfeeding (*n* = 92), did not complete the food frequency questionnaire (*n* = 22), provided implausible dietary reports (*n* = 205; see the *Statistical analysis* section), were excluded from this study. Additionally, one case of hypertriglyceridaemia (triglycerides> 8000 mg/dL) was excluded. The final sample consisted of 3524 adolescents (67.1% of the original sample) (Fig. 1). More details about the cohort can be found elsewhere [20, 21].

### Dietary assessment

A semi-quantitative food frequency questionnaire (FFQ) was administered in an electronic format [22]. Participants filled out the FFQ in a room with computers at the research clinic. A trained research assistant explained verbally and individually how subjects should use the platform before the FFQ was administered, even though the initial part of the platform presents the instructions on how to fill out the questionnaire. This aimed to minimize data entry errors and to maximize the quality of the data. For each food item, the participant was first asked about the frequency of intake and for those reported items the platform presents photographed images to identify the usual portion size (small, medium, or large). This electronic platform was developed to be used on the 18th year of follow up of the 1993 Pelotas Birth Cohort, and was based on a recent validation (paper-based FFQ) carried out when subjects from the 1993 Pelotas birth cohort were 15 years [23]. The electronic platform comprises all the food items included in the paper version of the FFQ [22]. The food items listed on the FFQ were based on a validated Brazilian FFQ [24] that was modified considering three 24-h recalls carried out in a sub-sample of the 1993 Pelotas Birth Cohort [23]. The energy intake (EI) ratio calculated based on the FFQ and the energy expenditure (EE) estimated by accelerometer was 1.13 [23]. The validation procedure did not consider macro or micronutrients.

The FFQ comprised 88 items, and eight frequency options were available: (i) five or more times per day; (ii) two to four times per day; (iii) once per day; (iv) five to six times per week; (v) two to four times per week; (vi) once per week; (vii) one to three times per month; and (viii) never or less than one time per month. Daily energy intake in kilocalories and nutrient intake were estimated using the Brazilian Food Composition Table [25], complemented with food items from the United States Department of Agriculture (USDA) National Nutrient Database for Standard Reference [26].

### Dietary patterns (main exposure)

FFQ items were combined into 45 food groups based on nutrient composition and frequency of consumption [27]. Those items consumed by 80% or more of the participants or when representative of the Brazilian dietary habits were considered separately (e.g., rice, beans, white bread, sugar, coffee, mate). Three alcoholic items (wine, beer and vodka) were excluded because this food group was consumed by less than 20% of the adolescents at age 18 [28, 29]. The 45 food groups are presented in the Additional file 1.

We applied principal component analysis (PCA) and rotation to derive dietary patterns [30]. A correlation matrix was constructed to assess the correlation between the food groups. The Kaiser-Meyer-Olkin test (≥0.8) and Bartlett’s test of sphericity (*p*-value < 0.05) were applied to verify whether the PCA assumptions were met [27, 29]. Varimax rotation was applied to obtain orthogonal factors. Food groups that showed factor loadings > 0.25 were considered to have strong associations with that factor. A positive factor loading indicated that the food group had a positive association with the pattern, while a negative factor loading indicated an inverse association [30]. Food groups were included in a dietary pattern if their factor loading was shown to be higher for that pattern in comparison to the others, even if these loadings were higher then 0.25 in other dietary patterns.

The number of factors that best represents the data was based on the scree plot and eigenvalues > 1.5 [27]. Dietary patterns were named according to the food items included and the dietary pattern interpretation. Participants received a factor score for each dietary pattern identified [28].

### Blood lipids (outcome variable)

A trained nurse was responsible for venous blood sample collection. Samples were kept for 30 min at ambient temperature and then centrifuged for 15 min at 2000 g. It was not possible to obtain fasting blood samples for logistic reasons, and the time of the last meal or snack consumed was registered for statistical adjustment. The average fasting time was 3 h and 20 min (95% CI 1 h – 12 h). Serum aliquots were stored at − 80 °C until analysis. Total cholesterol, HDL-cholesterol, LDL-cholesterol and triglycerides were measured using an automatic enzymatic colorimetric method with a Mindray BS-380 chemistry analyser (Shenzhen Mindray Bio-Medical Electronics Co., Ltd., China). The inter-assay coefficients of variation obtained for total cholesterol, HDL-cholesterol, LDL-cholesterol and triglycerides were 2.0%, 4.5%, 5.0% and 2.4%, respectively.

### Covariables

The following independent variables were included in the analyses: sex (girls/boys), skin colour (white, black/brown), total family income at birth (≤2, 3–5, ≥6 monthly minimum wage), maternal education at birth (< 8, ≥8 years of schooling completed), smoking habits at age 18 (no/yes), age of menarche (≤11; ≥12 years), leisure-time physical activity at age 18, and body mass index (BMI) at age 18.

BMI at age 18 was calculated using the weight measured to the nearest 0.1 kg using an electronic digital scale coupled with the BOD POD system (COSMED, Chicago, IL, USA) divided by the height measured to the nearest 0.1 cm using a portable stadiometer (C.M.S. Weighing Equipment Ltd., London, UK). The z-score of BMI for age and sex was calculated according to the WHO standards [31] and classified using the following cut-offs (BMI for age): underweight (≤ − 1 SD), normal weight (> − 1 SD and < + 1 SD), overweight (≥ + 1 SD and ≤ + 2 SD) and obese (> + 2 SD).

Physical activity was measured using the leisure-time information from the long version of the International Physical Activity Questionnaire (IPAQ) [32], which has been validated for the Brazilian population [33]. The questionnaire was administered to assess habitual physical activity. The number of days a week, and the amount of time spent per day on physical activities (including walking, moderate and vigorous physical activity) were recorded. Individuals who reported spending ≥300 min per week on physical activity were considered active [34].

### Statistical analysis

In order to test the association of each dietary patterns and blood lipids fraction, we first assessed the distribution of each lipid fraction to verify normality. We tested the differences in means or medians between categorical variables using Student’s *t* tests or Mann-Whitney U tests, ANOVA or Kruskal-Wallis tests, respectively, to evaluate the distribution of blood lipids according to each covariable.

Considering that misreporting of dietary intake may affect the associations between diet and non-communicable disease risk [35], we excluded subjects with implausible energy reports. The plausibility of each energy report was determined by comparing the reported EI with the estimate energy requirements (ER) [36, 37]. An energy report was considered implausible if the EI/ER ratio was outside ± 2 standard deviations range, and those subjects were excluded from the main analysis.

Dietary pattern scores were then categorized into tertiles, and the group with the lowest score was considered the reference category. Blood lipid fractions (total cholesterol, HDL-cholesterol, LDL-cholesterol and triglycerides) were considered as outcomes. The independent association between each dietary pattern and each lipid fraction was tested using crude and adjusted linear regressions. All regressions between dietary patterns and blood lipids were conducted separately for boys and girls. The independent effect of each dietary pattern was tested on each blood lipid fraction, adjusting for all variables with *p* value < 0.20 in the bivariate model. The adjusted variables were skin colour, maternal education at birth, total family income at birth, smoking habits at age 18, minutes of leisure-time physical activity per week at age 18, BMI at age 18, and age of menarche. Skin colour, maternal education at birth and total family income at birth were complementary socioeconomic measures to adjust for socioeconomic inequalities [20, 21]. The time elapsed since the last meal was included in every regression analysis to control for differences in non-fasting blood collection. In fully adjusted models, total EI was also included to adjust for differences in energy consumption among participants. Triglycerides were log-transformed due to their skewed distribution, and the beta coefficients of the crude and adjusted analyses are shown in their exponential form. The results should be interpreted as the % change (increase or decrease) in triglyceride level associated in relation to the reference group – i.e. a beta coefficient of 1.25 means 25% higher triglycerides in that group in relation to the reference one.

We performed further analyses of participants included and excluded in the current analyses to assess the effects of losses of follow up using chi-square test of proportions for all selected variables aiming to rule out selection bias. All statistical analyses were conducted using Stata 12.1 (Stata Corp., College Station, Texas, USA).

## Results

### Study participants

The baseline characteristics of the adolescents included in the current study differed from those considered losses to follow up according to sex, skin colour, maternal education at birth and total family income at birth (Additional file 2). At the 18 years follow up, those participants not included in the current study due to losses to follow up or presence of any exclusion criteria comprised a higher proportion of females (64.1%) compared to those that of the included sample (48.8%) (Additional file 3).

Of the 3524 adolescents included in the present analyses, 1718 were girls. Mean BMI of girls and boys were 23.3 ± 4.7 kg/m^2^ and 23.3 ± 4.1 kg/m^2^, respectively. Sixty-four per cent were white, and the majority lived with total family income ≤2 monthly minimum wages (60.2%). Twenty-six per cent were overweight or obese, and 13.6% declared themselves current smokers. Girls presented significantly higher mean/median lipid concentrations than boys. Differences in blood lipids were observed for all socioeconomic and lifestyle variables, age of menarche and BMI categories (Table 1). Differences in blood lipids according to the referred variables stratified by sex are presented in the Additional file 4.

**Table 1:** Mean/median (SD/IQR) blood lipids of adolescents at age 18 by selected characteristics. The 1993 Pelotas (Brazil) Birth Cohort

			Lipid profile (mg/dL)			
Variable	n	%	Total cholesterol	LDL cholesterol	HDL cholesterol	Triglycerides
			*P* value^*^ mean (SD)	*P* value^*^ mean (SD)	*P* value^*^ mean (SD)	*P* value^*^ median (IQR)
Sex			< 0.001	< 0.001	< 0.001	0.02
Girls	1718	48.7	170.8 (29.4)	94.5 (20.3)	60.2 (10.9)	71 (58; 91)
Boys	1806	51.3	152.8 (24.5)	84.4 (20.3)	51.7 (8.7)	68 (55; 89)
Skin colour^a^			< 0.001	< 0.001	0.032	< 0.001
White	2199	64.8	163.2 (29.1)	90.2 (23.2)	56.3 (11.1)	71 (58; 95)
Black/brown	1192	35.2	158.8 (27.5)	87.5 (22.1)	55.4 (10.2)	65 (54; 83)
Family income at birth (MMW)			< 0.001	0.007	< 0.001	< 0.001
≤ 2	2120	60.2	159.3 (28.0)	88.4 (22.8)	54.9 (10.3)	67 (56; 86)
3–5	843	23.9	163.8 (27.8)	90.4 (21.6)	56.5 (11.1)	71 (58; 94)
≥ 6	561	15.9	166.9 (30.3)	91.4 (24.2)	58.3 (11.3)	76 (60; 102)
Maternal education at birth (years)^b^			< 0.001	0.034	< 0.001	< 0.001
< 8	2129	60.5	159.6 (28.0)	88.7 (22.7)	54.7 (10.3)	68 (56; 88)
≥ 8	1389	39.5	164.5 (28.9)	90.3 (22.9)	57.5 (11.2)	72 (58; 96)
Smoking habit			< 0.001	0.008	< 0.001	0.530
No	3045	86.4	162.5 (28.4)	89.7 (22.6)	56.7 (10.8)	70 (57; 92)
Yes	479	13.6	155.7 (28.4)	86.8 (23.5)	52.7 (9.8)	69 (57; 89)
Leisure-time physical activity (min/week) < 0.001				< 0.001	< 0.001	0.008
< 300	1418	40.2	165.3 (28.5)	91.4 (22.9)	57.2 (10.8)	71 (58; 95)
≥ 300	2106	59.8	159.2 (28.2)	87.9 (22.6)	54.9 (10.7)	68 (56; 88)
Age of menarche (years)^c^			0.003	0.028	0.003	0.025
≤ 11	475	27.8	174.2 (30.4)	96.6 (24.4)	61.4 (10.9)	73 (59; 97)
≥ 12	1236	72.2	169.6 (29.0)	93.8 (23.9)	59.7 (10.9)	70 (58; 90)
Body mass index^d^			< 0.001	< 0.001	< 0.001	< 0.001
Under/normal weight	2585	73.3	159.0 (27.8)	86.9 (21.9)	56.5 (10.7)	67 (55; 85)
Overweight/obese	939	26.7	168.7 (29,0)	96.2 (23.8)	54.2 (10.7)	79 (61; 112)
Total N^*^	3524	100	161.6 (28.5)	89.3 (22.8)	55.8 (10.7)	69 (57; 91)

^*^P values refer to comparisons of means (SD) or medians (IQR) between groups using Student *t* tests or Mann-Whitney U tests, ANOVA or Kruskal-Wallis tests, respectively. ^a^133 missing values; ^b^6 missing values; ^c^7 missing values; ^d^BMI for age and sex reference in z score: under/normal weight ≤ + 1 SD; overweight/obese: > + 1SD. *Abbreviations: MMW* monthly minimum wages, *SD* Standard deviation, *IQR* Interquartile range

### Dietary patterns

Four dietary patterns were chosen to best describe adolescents’ food habits, and these explained 40% of the total variance. The first component had a high loading on all types of meat products, including processed meats (canned tuna/sardines, salt-cured meat, bacon) and fast foods (hamburgers, hot dogs) and was labelled the Meat Products and Fast Foods pattern. The second component loaded highly onto all types of fruits, vegetables and legumes (except black beans) and was labelled the Fruits and Vegetables pattern. The third component, labelled the Candies, Sodas and Dairy Products pattern, was characterized by high loads of chocolate powder, sugar-sweetened sodas, dairy products (milk, yogurt, hard cheese, soft cheese), sweets, chocolates, candies and caramels. The fourth component loaded highly onto black beans, white sugar, margarine/butter, white rice and white bread and was labelled the Common Brazilian Foods pattern (Table 2).

**Table 2:** Factor-loadings^a^ of food items for the major factors (dietary patterns) identified at the age of 18 based on a FFQ

Item	Food or food group	Meat Products and Fast Foods	Fruits and Vegetables	Candies, Sodas and Dairy Products	Common Brazilian Foods
1	Seafood	**0.3153**	0.0193	−0.0713	− 0.0962
2	Processed meats	**0.3135**	0.0057	−0.0238	0.0489
3	Pizza	**0.2999**	−0.0739	0.1104	−0.0884
4	Fast foods	**0.2898**	−0.0721	0.1391	−0.0274
5	Giblets	**0.2853**	−0.0189	−0.1368	0.0230
6	Chicken meat	**0.2715**	−0.0014	−0.0152	0.0567
7	Pork meat	**0.2581**	−0.0461	−0.0199	0.0930
8	Canned vegetables	0.2480	0.0077	−0.0661	−0.0318
9	Fried/baked salted pastries	0.2305	−0.0824	0.1460	0.0061
10	Popcorn	0.2097	0.0370	0.0219	0.0243
11	Pasta, polenta, corn	0.1923	0.1208	−0.0870	0.0245
12	French fries	0.1043	0.1107	0.0729	0.0418
13	Light soft drinks	0.1200	0.0675	0.0213	−0.0984
14	Banana	−0.1103	**0.3512**	0.1100	− 0.0207
15	Orange and tangerine	−0.0600	**0.3481**	0.0339	−0.0244
16	Other fruits	0.0272	**0.3591**	0.0420	−0.0959
17	Tomato	−0.1096	**0.3079**	−0.0190	0.1264
18	Other vegetables and legumes	0.1283	**0.3029**	0.1135	0.0056
19	Orange vegetables	0.1251	**0.2704**	−0.1214	−0.0464
20	Whole bread	0.0121	0.2026	−0.0602	−0.0249
21	Tubers	0.1123	0.1931	−0.0776	0.0522
22	Cake	0.0943	0.1775	0.0283	−0.0448
23	Fresh juice	0.0515	0.1530	0.1061	−0.0544
24	Chocolate powder	−0.0787	0.0439	**0.3786**	−0.0442
25	Sugar-sweetened sodas	0.0506	−0.1228	**0.3303**	0.0440
26	Milk and dairy products	−0.1062	0.2339	**0.3174**	−0.0964
27	Sweets and chocolates	0.1159	−0.0037	**0.2906**	−0.0151
28	Candies/caramels	−0.0051	− 0.0634	**0.2784**	−0.1327
29	Ice cream	0.0828	0.0023	**0.2639**	−0.0437
30	Mayonnaise	0.0380	0.0146	0.2222	0.0482
31	Sweet cookies	0.0215	0.0879	0.2016	0.0449
32	Luncheon meats	0.0179	0.0297	0.2008	0.1695
33	Snacks	0.1007	0.1103	0.1607	0.0216
34	Coffee	0.0280	−0.0647	−0.1131	**0.3977**
35	White sugar	−0.0179	− 0.1113	0.0966	**0.3901**
36	Black beans	−0.0150	0.0638	−0.0519	**0.3568**
37	Margarine and butter	0.0047	−0.0007	0.0726	**0.3513**
38	White rice	0.0028	0.0333	−0.0882	**0.2687**
39	White bread	−0.0841	0.0175	0.1077	**0.2516**
40	Vegetable spices	−0.0629	0.1842	−0.1193	0.2317
41	Red meats	0.1168	0.0335	0.0569	0.1696
42	Homemade bread	0.0719	0.0993	−0.1463	0.1553
43	Eggs	0.1231	0.0689	0.0154	0.1350
44	Artificial juice	0.0034	0.0447	0.0954	0.1340
45	“Mate” drink	−0.0425	0.0996	0.0265	0.1064
	**Explained variance (%)**	**14.5**	**10.3**	**7.5**	**7.7**

^a^Bold value means those items included in the pattern described in the column

### Dietary patterns and blood lipids

In the adjusted analyses, girls at the highest tertile of the Meat Products and Fast Foods pattern presented 1.5 mg/dL lower HDL-cholesterol compared with those at the lowest tertile. Girls at the second and third tertiles of the Candies, Sodas and Dairy Products pattern presented 5% and 10% higher triglycerides than girls in the first tertile, respectively. Girls at the second and third tertiles of the Common Brazilian Foods pattern presented 4 mg/dL lower total cholesterol compared with those classified in the first tertile. Additionally, girls classified in the second and third tertiles of the Common Brazilian Foods pattern had 6% lower triglycerides compared with those in the first tertile. The Fruits and Vegetables pattern was not associated with blood lipids (Table 3).

**Table 3:** Crude and adjusted linear regressions between dietary patterns and blood lipids among girls at age 18. The 1993 Pelotas (Brazil) Birth Cohort Study (*n* = 1718)

Dietary patterns	Total Cholesterol (mg/dL)		LDL Cholesterol (mg/dL)		HDL Cholesterol (mg/dL)		Triglycerides (mg/dL)^b^	
	Crude β (95% CI)	Adjusted^a^ β (95% CI)	Crude β (95% CI)	Adjusted^a^ β (95% CI)	Crude β (95% CI)	Adjusted^a^ β (95% CI)	Crude β^c^ (95% CI)	Adjusted^a^ β^c^ (95% CI)
Meat Products and Fast Foods								
1^st^ tertile	ref.	ref.	ref.	ref.	ref.	ref.	ref.	ref.
2^nd^ tertile	−0.84(− 4.25;2.56)	−1.04(− 4.47;2.38)	−0.25(− 3.04;2.53)	−0.47(− 3.29;2.34)	−0.65(− 1.91;0.61)	−0.75(− 1.99;0.49)	0.99(0.95;1.04)	0.99(0.96;1.04)
3^rd^ tertile	**−6.04(− 9.46;-2.61)**	−4.74(− 7.90;0.41)	− 2.69(− 5.49;0.11)	− 1.70(− 5.11;1.71)	**−2.54(− 3.81;-1.27)**	**−1.55(− 3.05;-0.04)**	**0.95(0.91;0.99)**	0.97(0.91;1.02)
Fruits and Vegetables								
1^st^ tertile	ref.	ref.	ref.	ref.	ref.	ref.	ref.	ref.
2^nd^ tertile	0.71(− 2.70;4.12)	0.85(− 2.61;4.31)	0.31(− 2.47;3.11)	0.09(− 2.75;2.92)	0.05(− 1.21;1.31)	0.40(− 0.85;1.65)	1.02(0.98;1.07)	1.02(0.98;1.07)
3^rd^ tertile	−2.15(− 5.58;1.27)	− 0.29(− 4.21;3.64)	− 0.30(− 3.10;2.49)	0.19(− 3.03;341)	**− 1.48(− 2.76;-0.22)**	−0.24(− 1.66;1.18)	0.98(0.93;1.02)	0.98(0.93;1.03)
Candies, Sodas and Dairy Products								
1^st^ tertile	ref.	ref.	ref.	ref.	ref.	ref.	ref.	ref.
2^nd^ tertile	0.31(−3.10;3.73)	0.89(−2.55;4.35)	−0.98(− 3.77;1.80)	−0.53(− 3.36;2.31)	0.90(− 0.37;2.17)	0.92(− 0.32;2.18)	1.04(0.99;1.09)	**1.05(1.01;1.09)**
3^rd^ tertile	− 0.64(− 4.06;2.78)	0.69(− 2.77;4.15)	−0.65(− 3.44;2.14)	2.59(− 0.99;6.17)	−0.01(− 1.28;1.26)	1.48(− 0.10;3.05)	1.03(0.99;1.08)	**1.10(1.05;1.16)**
Common Brazilian Foods								
1^st^ tertile	ref.	ref.	ref.	ref.	ref.	ref.	ref.	ref.
2^nd^ tertile	**−5.74(−9.14;-2.34)**	**−4.30(−7.75;-0.85)**	**− 4.08(−6.86;-1.30)**	**− 3.49(− 6.32;-0.66)**	−0.75(− 2.01;0.51)	0.29(− 0.98;1.56)	**0.92(0.88;0.97)**	**0.94(0.90;0.99)**
3^rd^ tertile	**−8.98(− 12.4;-5.58)**	**− 4.95(− 8.53;-1.36)**	**−5.21(− 7.99;-2.42)**	**−3.02(− 5.96;-0.08)**	**− 2.42(− 3.69;-1.15)**	−0.02(− 1.49;1.44)	**0.89(0.85;0.93)**	**0.93(0.89;0.98)**

^a^Adjusted for hours of fasting, skin colour, maternal education at birth, family income at birth, smoking habit at age 18, age of menarche, body mass index at age 18 and total energy intake at age 18.

^b^Logarithm transformation was used; ^c^β’s should be interpreted as the % change (increase or decrease) in triglyceride level associated in relation to the reference group

Bold values mean significant at *p* < 0.05.

Boys classified at the third tertile of the Common Brazilian Foods pattern presented 4.6 mg/dL and 3.8 mg/dL lower total and LDL-cholesterol, respectively, compared with those at the first tertile in the adjusted analysis. No associations were found between the Meat Products and Fast Foods; Fruits and Vegetables; or the Candies, Sodas and Dairy Products patterns and the blood lipids in the adjusted models for boys (Table 4).

**Table 4:** Crude and adjusted linear regressions between dietary pattern and blood lipids among boys at age 18. The 1993 Pelotas (Brazil) Birth Cohort Study (*n* = 1806)

Dietary patterns	Total Cholesterol (mg/dL)		LDL Cholesterol (mg/dL)		HDL Cholesterol (mg/dL)		Triglycerides (mg/dL)^b^	
	Crude β (95% CI)	Adjusted^a^ β (95% CI)	Crude β (95% CI)	Adjusted^a^ β (95% CI)	Crude β (95% CI)	Adjusted^a^ β (95% CI)	Crude β^c^ (95% CI)	Adjusted^a^ β^c^ (95% CI)
Meat Products and Fast Foods								
1^st^ tertile	ref.	ref.	ref.	ref.	ref.	ref.	ref.	ref.
2^nd^ tertile	− 1.07(− 3.84;1.70)	− 1.10(− 3.86;1.66)	−0.71(− 3.01;1.57)	−0.91(− 3.18;1.37)	0.05(− 0.94;1.05)	0.13(− 0.88;1.15)	0.97(0.93;1.02)	0.98(0.93;1.02)
3^rd^ tertile	**−3.26(− 6.04;-0.49)**	− 0.68(− 4.05;2.69)	**− 2.45(− 4.74;-0.16)**	−0.54(− 3.31;2.22)	−0.14(− 1.14;0.85)	0.21(− 1.02;1.46)	0.95(0.91;1.00)	0.98(0.93;1.04)
Fruits and Vegetables								
1^st^ tertile	ref.	ref.	ref.	ref.	ref.	ref.	ref.	ref.
2^nd^ tertile	0.39(− 2.39;3.16)	0.22(− 2.55;3.01)	0.60(− 1.69;2.89)	0.53(− 1.75;2.82)	0.47(− 0.52;1.46)	0.40(− 0.62;1.43)	0.96(0.91;1.00)	0.96(0.92;1.01)
3^rd^ tertile	− 1.99(− 4.76;0.79)	−1.04(− 4.38;2.30)	− 1.32(− 3.62;0.97)	− 0.90(− 3.64;1.85)	− 0.50(− 1.50;0.49)	−0.33(− 1.56;0.90)	0.98(0.94;1.03)	1.01(0.95;1.05)
Candies, Sodas and Dairy Products								
1^st^ tertile	ref.	ref.	ref.	ref.	ref.	ref.	ref.	ref.
2^nd^ tertile	0.96(− 2.35;4.27)	0.65(− 2.12;3.43)	0.71(−1.57;3.01)	0.70(− 1.58;2.98)	0.16(− 0.83;1.15)	− 0.00(− 1.03;1.02)	1.00(0.95;1.04)	1.00(0.96;1.04)
3^rd^ tertile	−0.74(− 4.07;2.58)	3.16(− 0.06;6.39)	− 0.18(− 2.48;2.10)	1.90(− 0.75;4.56)	0.81(− 0.18;1.80)	0.71(− 0.48;1.90)	1.01(0.97;1.06)	1.04(0.99;1.10)
Common Brazilian Foods								
1^st^ tertile	ref.	ref.	ref.	ref.	ref.	ref.	ref.	ref.
2^nd^ tertile	**−3.01(− 5.76;-0.26)**	−1.48(−4.28;1.32)	**− 2.38(− 4.66;-0.11)**	−1.41(− 3.71;0.89)	0.22(− 0.77;1.22)	0.25(− 0.78;1.29)	0.96(0.91;1.00)	0.99(0.94;1.03)
3^rd^ tertile	**−7.99(− 10.74;-5.24)**	**− 4.64(− 7.91;-1.37)**	**−6.21(− 8.49;-3.93)**	**−3.82(− 6.51;-1.13)**	− 0.76(− 1.75;0.23)	−0.91(− 2.12;0.30)	**0.94(0.89;0.98)**	1.02(0.96;1.07)

^a^Adjusted for hours of fasting, skin colour, maternal education at birth, family income at birth, smoking habits at age 18, minutes of leisure-time physical activity per week at age 18, body mass index at age 18, and total energy intake at age 18.

^b^Logarithm transformation was used; ^c^β’s should be interpreted as the % change (increase or decrease) in triglyceride level associated in relation to the reference group

Bold values mean significant at *P* < 0.05.

## Discussion

The dietary patterns derived in the present study that characterize the food consumption of 18-year-old adolescents from the 1993 Pelotas Birth Cohort were Meat Products and Fast Foods; Fruits and Vegetables; Candies, Sodas and Dairy Products; and Common Brazilian Foods. These patterns reflect the dietary behaviours of adolescents born in the nineties and are similar to those identified in the 1982 Pelotas Birth Cohort at 22 years, except that the Common Brazilian Foods pattern was the main pattern identified in the previous cohort [38], whereas in the current study, the Meat Products and Fast Foods pattern was dominant. This difference could be due, in part, to different age of the samples when foods were assessed; however differences may also reflect the increasing share of processed foods in the Brazilian diet over the last decades [12].

The dietary patterns identified in the present study are in line with other investigations of children and adolescents [9, 18, 19, 39, 40] including those reported by the Brazilian National School-based Health Survey [41]. The majority of these studies identified a Western or unhealthy pattern, usually characterized by fast foods, meat, sweets and candies, sugar-sweetened beverages, and a healthy or traditional pattern based on traditional foods, fruits and vegetables.

The Common Brazilian Foods pattern that emerged in the present study – albeit not as the main one – does reflect the maintenance of traditional foods in the adolescents diet, as “coffee, sugar, white bread and margarine/butter” and “white rice and black beans” that are part of the basic Brazilian breakfast and lunch [42], respectively. This dietary pattern was significantly associated with lower triglycerides among girls and lower total and LDL-cholesterol concentrations among both girls and boys. We highlighted that this dietary pattern was characterized by foods that may negatively impact blood lipids, as white sugar, margarine/butter, white rice and white bread [9, 10]. The significant association of this pattern with better blood lipids could be explained by the fact that the dietary pattern was constructed based on the frequency of consumption, rather than on the amount of food consumed. This pattern presented a high factor loading of beans, which are rich sources of insoluble fibre, and lower factor loading of foods rich in saturated fat, i.e., meat and dairy products. Although fruits and vegetables also represent foods rich in dietary fibre, black beans are a popular food item in Brazil [42]. It’s possible that adolescents that adhered most to this pattern consume a reasonable portion of beans rather than other foods rich in dietary fibre as fruits and vegetables.

The highest factor loading in the Meat Products and Fast Foods pattern was seafood products composed by fish and shrimp. However, the Pelotas market of fish are not those rich in omega-3 and fish is usually consumed fried, as a high frequency of this seafood is probably result of fried snack commonly eaten in restaurants and bars. The meat products and fast foods that also composed this pattern are rich sources of saturated fats. As a result, among girls, a high score on the Meat Products and Fast Foods pattern was associated with lower HDL-cholesterol. Our results are in line with other studies that reported a lower HDL-cholesterol among Iranian adolescents associated to red meat consumption [43], and the presence of metabolic syndrome in Brazilian adolescents associated to high consumption of ultra-processed foods [44]. Fast foods consumption among adolescents is commonly reported in studies that reinforce the need of promoting the adoption of healthy food choices [9].

The Candies, Sodas, and Dairy Products pattern was characterized by healthy and unhealthy foods. Milk and dairy products are a rich source of calcium, however milk is usually consumed with chocolate powder, and most dairy products are rich source of saturated fat, as well as yogurts are highly sweetened. This dietary pattern also presented a high factor loading on candies and sodas. The high score on the Candies, Sodas, and Dairy Products pattern was associated with higher triglycerides among girls. Sugar-sweetened beverages have been the focus of other studies, most of which show a negative impact on the cardiovascular health of children and adolescents [9]. A prospective study with Australian adolescents revealed a significant association between increased consumption of sugar-sweetened beverages over time and increased triglycerides in both sexes [45].

The Fruits and Vegetables pattern presented a high factor loading for all fruits and vegetables and a very low loading for red meat, indicating a vegetarian-like pattern. Although vegetarian diets are usually associated with better cardiometabolic parameters, such as low LDL-cholesterol and higher HDL-cholesterol [46], in the current study this pattern was not associated with blood lipids. However, adherence to a vegetarian pattern at this age is not indicative of positive health outcomes [47].

Few studies have investigated the associations between dietary patterns on blood lipids in adolescents [18, 19]. Other studies on this topic have included children or adults in the sample [48, 49], and results of these studies are inconsistent. A cluster analysis conducted by Song et al. [19] using data from three consecutive Korean surveys and a sample of 4347 adolescents observed no associations between any of the three dietary patterns identified (‘Western’, ‘traditional’ and ‘modified’) and elevated triglycerides (≥150 mg/dL) or low HDL-cholesterol (≤40 mg/dL), after adjusting for age and the year of the study. In a prospective Finnish cohort [18] composed of 1768 children and adolescents (aged 3 to 18 years), the traditional patterns based on sausages, potatoes, eggs, milk, butter, meats, rye, wheat, margarine and oil, and other cereals, was associated with higher total cholesterol, and LDL-cholesterol in both sexes. The health-conscious pattern based on fish and shellfish, legumes and nuts, root vegetables, fruits and berries, cheese, other dairy products, tea and alcoholic beverages presented no association with blood lipids. All analyses were adjusted for age, smoking status, physical activity and total energy intake [18]. On the other hand, a cross-sectional study conducted among 1214 Inupiat Eskimos (aged 18 to 92 years) observed an association between the traditional dietary pattern characterized by fish and oils, wild greens, stews made up mostly of meat, native meats and fruits and lower triglycerides concentrations. Meanwhile, the beverages and sweets dietary pattern was associated with higher LDL-cholesterol after adjusting for selected confounders [48]. A Canadian study of 635 participants (aged 18 to 55 years) observed that a Western pattern characterized by high consumption of refined grains, French fries and red meat was inversely correlated with LDL-cholesterol particle diameter after adjusting for age [49].

It is also important to discuss the public health implication of our finding. Following the cut-offs proposed by the American Guidelines for Cardiovascular Health and Risk Reduction in Children and Adolescents (10–19 years) [50], the prevalence of boderline/high blood lipids was 32.5% for total cholesterol (≥170 mg/dL), 15.7% for LDL-cholesterol (≥110 mg/dL), 16.2% for low HDL-cholesterol (≤45 mg/dL), and 20% for high triglycerides (≥100 mg/dL). These findings emphasize the need to promote healthy lifestyle and dietary habits early in life. We have previously shown that being active from adolescence to adulthood reduced the risk for high non-fasting triglycerides and current physical active was associated with greater HDL-cholesterol in young adults [51], and it is known that cardiovascular diseases usually manifest in the fourth decade of life, but atherosclerosis begins in the first years [50].

Girls exhibited higher blood lipid concentrations than boys. These findings are in line with the recent school-based Brazilian study of cardiovascular risks in adolescents that observed higher mean concentrations of total cholesterol, LDL-cholesterol and HDL-cholesterol among girls in comparison to boys [5]. Sex differences in blood lipids have also been reported in European studies of adolescents [52, 53]. Girls have more adipose tissue than boys, which may affect blood lipid concentrations [54]. Additionally, an age of menarche younger than 12 years was associated with higher blood lipids, which reflects hormonal conditions that adversely affect blood lipids independently of age and fat free mass [54]. We found that dietary patterns were more closely associated with blood lipids among girls in comparison to boys even after adjustments for socioeconomic and lifestyle factors, BMI and total energy intake.

The present study has some limitations that need to be highlighted. Regarding representativeness, despite of the reasonable sample size, the current study sample differed from targeted population regarding sex, skin colour, maternal education at birth, total family income at birth, smoking habit at age 18, and leisure-time physical activity at age 18. However, the magnitude of such differences is small, minimizing the likelihood of bias. Besides, analyses were stratified by sex and those variables were included in the adjusted models. Blood lipids were collected without fasting for logistical reasons. However, there is no evidence that fasting blood samples are superior to non-fasting blood samples to assay blood lipids for cardiovascular risk assessment in primary prevention [55]. Non-fasting blood better reflects the daily mean plasma lipoprotein concentrations because most individuals consume several meals during the day, and therefore, the postprandial state predominates a 24-h period [55]. In the current study, LDL-cholesterol was directly measured and this lipid fraction does not change with fasting [55, 56]. Triglycerides levels differ on the basis of fasting status, and a systematic difference within subjects according to the time elapsed since the last meal is possible [55, 56]. Thus, hours of fasting were included in all the adjusted models to adjust for this difference. Even though, results should be interpreted with caution. The administered FFQ was developed for the follow-up at age 15 of the 1993 Birth Cohort [23] and was based on a validated FFQ [24], but it was not validated for the follow-up at age 18. We cannot rule out the possibility of misreporting of food consumption. Factor analyses depend on several decisions made by the researcher, such as food group combinations and the number of factors retained. This statistical procedure allows different input of variables to derive dietary patterns (i.e. frequency, grams or portion size) with or without adjustment for total energy intake before the dietary patterns are established. The classification of individuals according to scores in each pattern may allow some individuals comply equally in different patterns. In the current study, we performed the PCA based on frequency of consumption and four meaningful dietary patterns were obtained with factor loading of individual food items very distinct across major patterns. In addition, this is a qualitative analysis rather than a quantitative one, and it cannot be interpreted in the same way as conventional dietary approaches. Another limitation stems from the cross-sectional nature of the analyses and the possibility of reverse causality, as dietary patterns and blood lipids were both measured at age 18. In spite of these limitations, our study has important strengths, such as the large sample size from a population-based study, the high response rate and the application of standard questionnaires.

## Conclusion

The present study showed associations between dietary patterns and blood lipids in Brazilian adolescents followed from birth to age 18. Blood lipids were more closely associated with dietary patterns among girls than among boys. However, higher scores for the Common Brazilian Foods pattern were associated with lower total cholesterol for both girls and boys. It is possible that the negative effects of an unhealthy dietary pattern manifest later in life. Our results emphasize the importance of understanding perceptions of dietary patterns among adolescents and of efforts to include traditional foods in their diets.

## Additional files

Additional file 1: Forty-five food items of the food frequency questionnaire categorized according to nutrient composition, frequency of consumption or traditional habits. The 1993 Pelotas (Brazil) Birth Cohort. (DOCX 19 kb)

Additional file 2: Frequency distribution of baseline characteristics of participants included and excluded from the present analyses. The 1993 Pelotas (Brazil) Birth Cohort. (DOCX 16 kb)

Additional file 3: Frequency distribution of main investigated variables at age 18 between those who were included and excluded from the present analyses. The 1993 Pelotas (Brazil) Birth Cohort. (DOCX 17 kb)

Additional file 4: Mean/median (SD/IQR) blood lipids of adolescents at age 18 by selected characteristics. The 1993 Pelotas (Brazil) Birth Cohort. (DOCX 19 kb)

## Abbreviations

BMI: Body mass index
CI: Confidence interval
EI: Energy intake
ER: Energy requirements
FFQ: Food frequency lipoprotein
HDL: High density lipoprotein
LDL: Low density lipoprotein
PCA: Principal component analysis

## References

[1] De Henauw S, Michels N, Vyncke K, Hebestreit A, Russo P, Intemann T (2014). Blood lipids among young children in Europe: results from the European IDEFICS study. Int J Obes.

[2] Khoury M, Manlhiot C, Gibson D, Chahal N, Stearne K, Dobbin S (2016). Universal screening for cardiovascular disease risk factors in adolescents to identify high-risk families: a population-based cross-sectional study. BMC Pediatr..

[3] Juhola J, Magnussen CG, Viikari JS, Kahonen M, Hutri-Kahonen N, Jula A (2011). Tracking of serum lipid levels, blood pressure, and body mass index from childhood to adulthood: the Cardiovascular Risk in Young Finns Study. J Pediatr..

[4] Nicklas TA, von Duvillard SP, Berenson GS (2002). Tracking of serum lipids and lipoproteins from childhood to dyslipidemia in adults: the Bogalusa Heart Study. Int J Sports Med..

[5] Faria Neto JR, Bento VF, Baena CP, Olandoski M, Goncalves LG, Abreu Gde A (2016). ERICA: prevalence of dyslipidemia in Brazilian adolescents. Rev. Saude Publica.

[6] Kelishadi R, Haghdoost AA, Moosazadeh M, Keikha M, Aliramezany M (2015). A systematic review and meta-analysis on screening lipid disorders in the pediatric age group. J Res Med Sci..

[7] Kim SH, Song YH, Park S, Park MJ (2016). Impact of lifestyle factors on trends in lipid profiles among Korean adolescents: the Korea National Health and Nutrition Examination Surveys study, 1998 and 2010. Korean J Pediatr..

[8] Ordovas JM (2009). Genetic influences on blood lipids and cardiovascular disease risk: tools for primary prevention. Am J Clin Nutr..

[9] Funtikova AN, Navarro E, Bawaked RA, Fito M, Schroder H (2015). Impact of diet on cardiometabolic health in children and adolescents. Nutr J..

[10] Siri-Tarino PW, Krauss RM (2016). Diet, lipids, and cardiovascular disease. Curr Opin Lipidol..

[11] Monteiro CA, Cannon G, Moubarac JC, Martins AP, Martins CA, Garzillo J (2015). Dietary guidelines to nourish humanity and the planet in the twenty-first century. A blueprint from Brazil. Public Health Nutr..

[12] Monteiro CA, Levy RB, Claro RM, de Castro IR, Cannon G (2011). Increasing consumption of ultra-processed foods and likely impact on human health: evidence from Brazil. Public Health Nutr..

[13] Canella DS, Levy RB, Martins AP, Claro RM, Moubarac JC, Baraldi LG (2014). Ultra-processed food products and obesity in Brazilian households (2008–2009). PloS One..

[14] Bel-Serrat S, Mouratidou T, Huybrechts I, Labayen I, Cuenca-Garcia M, Palacios G (2014). Associations between macronutrient intake and serum lipid profile depend on body fat in European adolescents: the Healthy Lifestyle in Europe by Nutrition in Adolescence (HELENA) study. Br J Nutr..

[15] Bradlee ML, Singer MR, Daniels SR, Moore LL (2013). Eating patterns and lipid levels in older adolescent girls. Nutr Metab Cardiovasc Dis..

[16] Tapsell LC, Neale EP, Satija A, Hu FB (2016). Foods, Nutrients, and Dietary Patterns: Interconnections and Implications for Dietary Guidelines. Adv Nutr..

[17] Joung H, Hong S, Song Y, Ahn BC, Park MJ (2012). Dietary patterns and metabolic syndrome risk factors among adolescents. Korean J Pediatr..

[18] Mikkila V, Rasanen L, Raitakari OT, Marniemi J, Pietinen P, Ronnemaa T (2007). Major dietary patterns and cardiovascular risk factors from childhood to adulthood. The Cardiovascular Risk in Young Finns Study. Br J Nutr..

[19] Song Y, Park MJ, Paik HY, Joung H (2010). Secular trends in dietary patterns and obesity-related risk factors in Korean adolescents aged 10–19 years. Int J Obes..

[20] Victora CG, Araujo CL, Menezes AM, Hallal PC, Vieira Mde F, Neutzling MB (2006). Methodological aspects of the 1993 Pelotas (Brazil) Birth Cohort Study. Rev. Saude Publica..

[21] Goncalves H, Assuncao MC, Wehrmeister FC, Oliveira IO, Barros FC, Victora CG (2014). Cohort profile update: The 1993 Pelotas (Brazil) birth cohort follow-up visits in adolescence. Int J Epidemiol..

[22] Schneider BC, Motta JVS, Muniz LC, Bielemann RM, Madruga SW, Orlandi SP (2016). Design of a digital and self-reported food frequency questionnaire to estimate food consumption in adolescents and young adults: birth cohorts at Pelotas, Rio Grande do Sul, Brazil. Rev. Bras Epidemiol.

[23] Gigante DP, Reichert FF, Hallal PC, Souza RV, Neutzling MB, Vieira Mde F (2010). Dietary assessment in the 1993 Pelotas (Brazil) birth cohort study: comparing energy intake with energy expenditure. Cad Saude Publica..

[24] Sichieri R, Everhart JE (1998). Validity of a Brazilian food frequency questionnaire against dietary recalls and estimated energy intake. Nutr Res..

[25] Tabela Brasileira de Composição de Alimentos. Universidade Estadual de Campinas - NEPA/UNICAMP. 2011. http://www.nepa.unicamp.br/taco/index.php. Accessed 10 Nov 2017.

[26] USDA National Nutrient Database for Standard Reference, Release 24. U.S. Department of Agriculture, Agriculture Research Service. 2011. http://ars.usda.gov. Accessed 10 Nov 2017.

[27] Newby PK, Tucker KL (2004). Empirically derived eating patterns using factor or cluster analysis: a review. Nutr Rev..

[28] Hu FB (2002). Dietary pattern analysis: a new direction in nutritional epidemiology. Curr Opin Lipidol..

[29] Wirfalt E, Drake I, Wallstrom P. What do review papers conclude about food and dietary patterns? Food Nutr Res. 2013;57. 10.3402/fnr.v57i0.20523. 10.3402/fnr.v57i0.20523 PMC3589439 23467387

[30] Northstone K, Emmett P, Rogers I (2008). Dietary patterns in pregnancy and associations with socio-demographic and lifestyle factors. Eur J Clin Nutr..

[31] de Onis M, Onyango AW, Borghi E, Siyam A, Nishida C, Siekmann J (2007). Development of a WHO growth reference for school-aged children and adolescents. Bull World Health Organ..

[32] Hagstromer M, Oja P, Sjostrom M (2006). The International Physical Activity Questionnaire (IPAQ): a study of concurrent and construct validity. Public Health Nutr..

[33] Matsudo S, Araujo T, Matsudo V, Oliveira L, D. A, E. A (2001). Questionário Internacional de Atividade Física (IPAQ): Estudo de Validade e Reprodutibilidade no Brasil. Rev. Bras Ativ Saude.

[34] Global Recommendations on Physical Activity for Health. WHO. World Health Organization, Genebra. 2010. http://www.who.int/dietphysicalactivity/publications/9789241599979/en/ Acessed 10 Nov 2017. 26180873

[35] Jenab M, Slimani N, Bictash M, Ferrari P, Bingham SA (2009). Biomarkers in nutritional epidemiology: applications, needs and new horizons. Hum Genet..

[36] Huang TT, Howarth NC, Lin BH, Roberts SB, McCrory MA (2004). Energy intake and meal portions: associations with BMI percentile in U.S. children. Obes Res..

[37] McCrory MA, McCrory MA, Hajduk CL, Roberts SB (2002). Procedures for screening out inaccurate reports of dietary energy intake. Public Health Nutr..

[38] Olinto MT, Willett WC, Gigante DP, Victora CG (2011). Sociodemographic and lifestyle characteristics in relation to dietary patterns among young Brazilian adults. Public Health Nutr..

[39] Ambrosini GL, Huang RC, Mori TA, Hands BP, O’Sullivan TA, de Klerk NH (2010). Dietary patterns and markers for the metabolic syndrome in Australian adolescents. Nutr Metab Cardiovasc Dis..

[40] Nobre LN, Lamounier JA, Franceschini SC (2013). Sociodemographic, anthropometric and dietary determinants of dyslipidemia in preschoolers. J Pediatr..

[41] Tavares LF, Castro IR, Levy RB, Cardoso Lde O, Claro RM (2014). Dietary patterns of Brazilian adolescents: results of the Brazilian National School-Based Health Survey (PeNSE). Cad Saude Publica..

[42] Md S, Brasil (2014). Cadernos de atenção básica. Guia alimentar para a população brasileira.

[43] Kelishadi R, Pour MH, Zadegan NS, Kahbazi M, Sadry G, Amani A (2004). Dietary fat intake and lipid profiles of Iranian adolescents: Isfahan Healthy Heart Program--Heart Health Promotion from Childhood. Prev Med..

[44] Tavares LF, Fonseca SC, Garcia Rosa ML, Yokoo EM (2012). Relationship between ultra-processed foods and metabolic syndrome in adolescents from a Brazilian Family Doctor Program. Public Health Nutr..

[45] Ambrosini GL, Oddy WH, Huang RC, Mori TA, Beilin LJ, Jebb SA (2013). Prospective associations between sugar-sweetened beverage intakes and cardiometabolic risk factors in adolescents. Am J Clin Nutr..

[46] Sabate J, Wien M (2010). Vegetarian diets and childhood obesity prevention. Am J Clin Nutr..

[47] Robinson-O’Brien R, Perry CL, Wall MM, Story M, Neumark-Sztainer D (2009). Adolescent and young adult vegetarianism: better dietary intake and weight outcomes but increased risk of disordered eating behaviors. J Am Diet Assoc..

[48] Eilat-Adar S, Mete M, Nobmann ED, Xu J, Fabsitz RR, Ebbesson SO (2009). Dietary patterns are linked to cardiovascular risk factors but not to inflammatory markers in Alaska Eskimos. J Nutr..

[49] Bouchard-Mercier A, Paradis AM, Godin G, Lamarche B, Perusse L, Vohl MC (2010). Associations between dietary patterns and LDL peak particle diameter: a cross-sectional study. J Am Coll Nutr..

[50] National Heart Lung and Blood Institute (2012). Expert Panel on Integrated Guidelis for Cardiovascular Health and Risk Reduction in Children and Adolescents Summary Report. NIH Publication No. 12-7486A.

[51] Bielemann RM, Ramires VV, Gigante DP, Hallal PC, Horta BL (2014). Longitudinal and cross-sectional associations of physical activity with triglyceride and HDLc levels in young male adults. J Phys Act Health..

[52] Spinneker A, Egert S, Gonzalez-Gross M, Breidenassel C, Albers U, Stoffel-Wagner B (2012). Lipid, lipoprotein and apolipoprotein profiles in European adolescents and its associations with gender, biological maturity and body fat--the HELENA Study. Eur J Clin Nutr..

[53] Ucar B, Kilic Z, Dinleyici EC, Colak O, Gunes E (2007). Serum lipid profiles including non-high density lipoprotein cholesterol levels in Turkish school-children. Anadolu Kardiyol Derg..

[54] Remsberg KE, Demerath EW, Schubert CM, Chumlea WC, Sun SS, Siervogel RM (2005). Early menarche and the development of cardiovascular disease risk factors in adolescent girls: the Fels Longitudinal Study. J Clin Endocrinol Metab..

[55] Nordestgaard BG, Langsted A, Mora S, Kolovou G, Baum H, Bruckert E (2016). Fasting Is Not Routinely Required for Determination of a Lipid Profile: Clinical and Laboratory Implications Including Flagging at Desirable Concentration Cutpoints-A Joint Consensus Statement from the European Atherosclerosis Society and European Federation of Clinical Chemistry and Laboratory Medicine. Clin Chem..

[56] Steiner MJ, Skinner AC, Perrin EM (2011). Fasting might not be necessary before lipid screening: a nationally representative cross-sectional study. Pediatrics..

