# Supplementary material for: Dietary patterns are associated with blood lipids at 18-year-olds: a cross-sectional analysis nested in the 1993 Pelotas (Brazil) birth cohort
Source: Nutr J. 2018 Aug 22;17:77. doi: 10.1186/s12937-018-0389-z (PMC6106900; doi:10.1186/s12937-018-0389-z)
Supplement: Supplementary file 1 — Forty-five food items of the food frequency questionnaire categorized according to nutrient composition, frequency of consumption or traditional habits. The 1993 Pelotas (Brazil) Birth Cohort. (DOCX 19 kb) [file 12937_2018_389_MOESM1_ESM.docx]

**Additional file 1:** Forty-five food items of the food frequency questionnaire categorized according to nutrient composition, frequency of consumption or traditional habits. The 1993 Pelotas (Brazil) Birth Cohort.

| **Item** | **Food item** | **Item** | **Food item** |
| --- | --- | --- | --- |
| 1 | Rice | 24 | Luncheon meats *(ham, mortadella/sausage)* |
| 2 | Black beans | 25 | Eggs |
| 3 | Coffee | 26 | Dairy products *(milk, yogurt, hard cheese, soft cheese)* |
| 4 | Sugar | 27 | Fast foods *(hamburgers, hot dogs)* |
| 5 | White bread | 28 | Fried/baked salted pastries |
| 6 | Homemade bread | 29 | Pizza |
| 7 | Whole bread | 30 | French fries |
| 8 | Tubers *(potato, cassava)* | 31 | Fat *(butter, margarine)* |
| 9 | Other cereals *(pasta, fried polenta, corn)* | 32 | Mayonnaise |
| 10 | Tomato | 33 | Fresh juice |
| 11 | Vegetable spices *(garlic, onion, sweet pepper)* | 34 | Artificial fruit juice |
| 12 | Orange vegetables *(carrot, pumpkin)* | 35 | Sugar-sweetened sodas |
| 13 | Other vegetables and legumes *(cabbage, lettuce, chayote, cucumber, green beans, beetroot, cauliflower, lentil, pea, chickpea)* | 36 | Artificially-sweetened sodas |
| 14 | Canned vegetables | 37 | “Mate” drink |
| 15 | Banana | 38 | Chocolate powder |
| 16 | Orange or tangerine | 39 | Candies/caramels |
| 17 | Other fruits *(papaya, pineapple, avocado, mango, peach, guava, pear, apple, watermelon or melon, strawberry, grape)* | 40 | Sweets *(pudding/desserts, chocolate bar)* |
| 18 | Red meats *(steak, t-bone beef)* | 41 | Cake |
| 19 | Pork meat | 42 | Ice cream |
| 20 | Seafoods *(fish, shrimp)* | 43 | Sweet cookies |
| 21 | Chicken meats *(roast, fried)* | 44 | Snacks *(chips, salty crackers)* |
| 22 | Giblets *(gizzard, heart, liver)* | 45 | Popcorn |
| 23 | Processed meats *(canned, salt-cured meat, bacon)* |  |  |
