# Supplementary material for: Dietary patterns are associated with blood lipids at 18-year-olds: a cross-sectional analysis nested in the 1993 Pelotas (Brazil) birth cohort
Source: Nutr J. 2018 Aug 22;17:77. doi: 10.1186/s12937-018-0389-z (PMC6106900; doi:10.1186/s12937-018-0389-z)
Supplement: Supplementary file 2 — Frequency distribution of baseline characteristics of participants included and excluded from the present analyses. The 1993 Pelotas (Brazil) Birth Cohort. (DOCX 16 kb) [file 12937_2018_389_MOESM2_ESM.docx]

**Additional file 2:** Frequency distribution of baseline characteristics of participants included and excluded from the present analyses. The 1993 Pelotas (Brazil) Birth Cohort.

| Variable | Original N (1993) | Included in the present analyses | | Not included in the present analyses^†^ | | *P*  value* |
| --- | --- | --- | --- | --- | --- | --- |
|  |  | n | % | n | % |  |
| **Sex** |  |  |  |  |  |  |
| girls | 2,645 | 1,718 | 48.8 | 927 | 53.8 | 0.001 |
| boys | 2,603 | 1,806 | 51.2 | 797 | 46.2 |  |
| **Skin colour** |  |  |  |  |  |  |
| white | 2,769 | 2,199 | 64.9 | 570 | 61.2 | 0.038 |
| black/brown | 1,554 | 1,192 | 35.1 | 362 | 38.8 |  |
| **Family income at birth (MMW)** | | |  |  |  |  |
| ≤2 | 3,227 | 2,120 | 60.2 | 1,107 | 64.2 |  |
| 3-5 | 1,204 | 843 | 23.9 | 361 | 20.9 | 0.015 |
| ≥6 | 818 | 561 | 15.9 | 257 | 14.9 |  |
| **Maternal education at birth (years)** | | |  |  |  |  |
| < 8 | 3,230 | 2,129 | 60.5 | 1,101 | 63.8 | 0.019 |
| ≥ 8 | 2,012 | 1,389 | 39.5 | 623 | 36.2 |  |
| *Total N* | 5,249 | 3,524 | 67.1 | 1724 | 32.9 |  |

* *P*-values refer to chi-square test for comparisons;

MMW: monthly minimum wages

^†^ Not included those participants that were considered losses at the 18 year follow-up (n=979), presented missing data or exclusion criteria as follow: have refused to provide blood samples (n=180), were pregnant (n=57) and breastfeeding (n=92), have reported use of medication for glucose or lipid control [insulin (n=9), metformin (n=2), statins (n=2)], presented haemoglobin A1c ≥6.5% (n=12), did not complete the food frequency questionnaire (n=22), provided implausible dietary reports (n=205), one case of hypertriglyceridaemia (triglycerides>8,000).
