# Supplementary material for: Dietary patterns are associated with blood lipids at 18-year-olds: a cross-sectional analysis nested in the 1993 Pelotas (Brazil) birth cohort
Source: Nutr J. 2018 Aug 22;17:77. doi: 10.1186/s12937-018-0389-z (PMC6106900; doi:10.1186/s12937-018-0389-z)
Supplement: Supplementary file 3 — Frequency distribution of main investigated variables at age 18 between those who were included and excluded from the present analyses. The 1993 Pelotas (Brazil) Birth Cohort. (DOCX 17 kb) [file 12937_2018_389_MOESM3_ESM.docx]

**Additional file 3:** Frequency distribution of main investigated variables at age 18 between those who were included and excluded from the present analyses. The 1993 Pelotas (Brazil) Birth Cohort.

| Variable | Included in the present analyses | | Excluded from the present analyses^†^ | | *P*  value* |
| --- | --- | --- | --- | --- | --- |
|  | n | % | n | % |  |
| **Sex** |  |  |  |  |  |
| girls | 1,718 | 48.8 | 373 | 64.1 | <0.001 |
| boys | 1,806 | 51.2 | 209 | 35.9 |  |
| **Skin colour** |  |  |  |  |  |
| white | 2,199 | 64.9 | 327 | 60.2 | 0.037 |
| black/brown | 1,192 | 35.1 | 216 | 39.8 |  |
| **Family income at birth (MMW)** | | |  |  |  |
| ≤2 | 2,120 | 60.2 | 377 | 64.8 |  |
| 3-5 | 843 | 23.9 | 142 | 24.4 | 0.006 |
| ≥6 | 561 | 15.9 | 63 | 10.8 |  |
| **Maternal education at birth (years)** | | |  |  |  |
| < 8 | 2,129 | 60.5 | 386 | 66.4 | 0.007 |
| ≥ 8 | 1,389 | 39.5 | 195 | 33.6 |  |
| **Smoking habit** |  |  |  |  |  |
| No | 3,045 | 86.4 | 479 | 82.4 | 0.011 |
| Yes | 479 | 13.6 | 102 | 17.6 |  |
| **Leisure-time physical activity (minutes/week)** | | |  |  |  |
| < 300 | 1,418 | 40.2 | 256 | 44.8 | 0.038 |
| ≥ 300 | 2,106 | 59.8 | 315 | 55.2 |  |
| **Age of menarche (years)** | | |  |  |  |
| ≤ 11 | 475 | 27.8 | 102 | 27.6 | 0.940 |
| ≥12 | 1,236 | 72.4 | 268 | 72.4 |  |
| **Body mass index**^1^ |  |  |  |  |  |
| Underweight | 47 | 1.3 | 6 | 1.4 |  |
| Normal weight | 2,538 | 72.0 | 290 | 66.3 | 0.100 |
| Overweight | 591 | 16.7 | 89 | 20.4 |  |
| Obese | 348 | 10.0 | 52 | 11.9 |  |
| *Total N^2^* | 3,524 |  | 582 |  |  |

* *P*-values refer to chi-square test for comparisons; MMW: monthly minimum wages. ^1^ BMI for age and sex reference in z score: underweight (≤ -1 SD), normal weight (> -1 SD and < +1 SD), overweight (≥ +1SD and ≤ +2 SD), obese (> +2 SD). ^2^ Included and not included in the present study represents 4,106 adolescents interviewed at 18 year follow-up; adding to those known to have died (n=164), it represents a retention rate of 81.3%. ^†^ Missing data or exclusion criteria to the present analyses: refused to provide blood samples (n=180), pregnancy (n=57) and breastfeeding (n=92), reported use of medication for glucose or lipid control [insulin (n=9), metformin (n=2), statins (n=2)], had haemoglobin A1c ≥6.5% (n=12), did not complete the food frequency questionnaire (n=22), provided implausible dietary reports (n=205), one case of hypertriglyceridaemia (triglycerides>8,000).
