# Supplementary material for: Dietary patterns are associated with blood lipids at 18-year-olds: a cross-sectional analysis nested in the 1993 Pelotas (Brazil) birth cohort
Source: Nutr J. 2018 Aug 22;17:77. doi: 10.1186/s12937-018-0389-z (PMC6106900; doi:10.1186/s12937-018-0389-z)
Supplement: Supplementary file 4 — Mean/median (SD/IQR) blood lipids of adolescents at age 18 by selected characteristics. The 1993 Pelotas (Brazil) Birth Cohort. (DOCX 19 kb) [file 12937_2018_389_MOESM4_ESM.docx]

**Additional file 4:** Mean/median (SD/IQR) blood lipids of adolescents at age 18 by selected characteristics. The 1993 Pelotas (Brazil) Birth Cohort.

|  |  | **Lipid profile (mg/dL)** | | | | | | | | |
| --- | --- | --- | --- | --- | --- | --- | --- | --- | --- | --- |
|  | **Girls** | | | | | **Boys** | | | | |
| Variable | n | Total cholesterol | LDL cholesterol | HDL cholesterol | Triglycerides | n | Total cholesterol | LDL cholesterol | HDL cholesterol | Triglycerides |
|  |  | *P* value^1^  mean (SD) | *P* value^1^  mean (SD) | *P* value^1^  mean (SD) | *P* value^1^  median (IQR) |  | *P* value^1^  mean (SD) | *P* value^1^  mean (SD) | *P* value^1^  mean (SD) | *P* value^1^  median (IQR) |
| ***Total N*** | 1,718 | 170.8 (29.4) | 94.5 (24.1) | 60.1 (10.9) | 71 (58-93) | 1,806 | 152.8 (24.5) | 84.4 (20.3) | 51.7 (8.7) | 68 (55-89) |
| **Skin colour**^2^ |  | <0.001 | 0.019 | <0.001 | <0.001 |  | 0.028 | 0.021 | 0.283 | <0.001 |
| white | 1,100 | 172.9 (30.1) | 95.5 (24.2) | 60.9 (11.3) | 73 (59-97) | 1,099 | 153.5 (24.4) | 84.9 (20.8) | 51.6 (8.7) | 70 (57-93) |
| black/brown | 584 | 167.3 (27.8) | 92.6 (23.7) | 58.9 (10.2) | 66 (55-83) | 608 | 150.8 (24.9) | 82.6 (19.3) | 52.1 (8.9) | 65 (54-83) |
| **Family income at birth (MMW)** | | <0.001 | 0.161 | <0.001 | <0.001 |  | <0.001 | 0.059 | <0.001 | 0.022 |
| ≤2 | 1,012 | 168.4 (28.9) | 93.8 (24.2) | 58.9 (10.5) | 68 (57-87) | 1,108 | 151.1 (24.4) | 83.5 (20.2) | 51.2 (8.6) | 66 (54-86) |
| 3-5 | 424 | 172.4 (28.5) | 94.9 (22.7) | 60.8 (11.6) | 73 (59-96) | 419 | 155.1 (24.1) | 85.8 (19.4) | 52.2 (8.5) | 69 (56-91) |
| ≥6 | 282 | 177.2 (31.7) | 96.8 (25.4) | 63.4 (10.8) | 79 (62-109) | 279 | 156.5 (24.8) | 85.8 (21.6) | 53.2 (9.4) | 74 (59-98) |
| **Maternal education at birth (years)**^3^ | | <0.001 | 0.181 | <0.001 | 0.003 |  | <0.001 | 0.050 | 0.001 | <0.001 |
| < 8 | 1,046 | 168.4 (28.8) | 93.9 (24.1) | 58.4 (10.5) | 70 (58-90) | 1,083 | 151.2 (24.4) | 83.6 (19.9) | 51.2 (8.6) | 66 (54-86) |
| ≥ 8 | 669 | 174.6 (30.0) | 95.5 (23.9) | 62.8 (11.1) | 72 (59-99) | 720 | 155.2 (24.4) | 85.5 (20.8) | 52.6 (8.8) | 71 (58-94) |
| **Smoking habit** |  | 0.006 | 0.628 | <0.001 | 0.852 |  | <0.001 | 0.007 | <0.001 | 0.434 |
| No | 1,509 | 171.6 (29.7) | 94.6 (24.2) | 60.7 (10.9) | 71 (58-93) | 1,536 | 153.7 (23.9) | 84.9 (19.9) | 52.1 (8.7) | 68 (55-89) |
| Yes | 209 | 165.7 (26.9) | 93.8 (23.2) | 56.1 (10.2) | 70 (58-93) | 270 | 147.9 (27.1) | 81.4 (22.3) | 50.1 (8.5) | 67 (55-86) |
| **Leisure-time physical activity (min/week)** 0.448 | | | 0.624 | 0.993 | 0.463 |  | 0.139 | 0.289 | 0.773 | 0.040 |
| < 300 | 981 | 171.3 (28.5) | 94.8 (23.6) | 60.1 (10.6) | 71 (58-95) | 497 | 154.2 (24.8) | 85.2 (20.2) | 51.6 (8.7) | 70 (56-95) |
| ≥ 300 | 797 | 170.3 (30.4) | 94.2 (24.5) | 60.1 (11.4) | 70 (58-90) | 1,309 | 152.3 (24.4) | 84.1 (20.3) | 51.7 (8.7) | 67 (55-88) |
| **Body mass index^4^** | | 0.047 | <0.001 | <0.001 | <0.001 |  | <0.001 | <0.001 | <0.001 | <0.001 |
| Under/normal weight | 1,225 | 169.9 (29.6) | 93.3 (24.0) | 60.9 (10.9) | 69 (57-88) | 1,360 | 149.2 (21.9) | 81.1 (17.8) | 52.4 (8.7) | 65 (54-83) |
| Overweight/obese | 493 | 173.1 (28.9) | 97.8 (23.8) | 58.1 (10.9) | 75 (60-101) | 446 | 163.9 (28.4) | 94.4 (23.7) | 49.7 (8.4) | 83 (62-122) |
| **Age of menarche (years)**^5^ | | 0.003 | 0.028 | 0.003 | 0.042 |  |  |  |  |  |
| ≤ 11 | 475 | 174.2 (30.4) | 96.6 (24.4) | 61.4 (10.9) | 73 (59-97) | - | - | - | - | - |
| ≥12 | 1,236 | 169.6 (29.0) | 93.7 (23.9) | 59.7 (10.9) | 70 (58-90) | - | - | - | - | - |

^1^ *P* values refer to comparisons of means (SD) or medians (IQR) between groups using Student *t* tests or Mann-Whitney U tests, ANOVA or Kruskal-Wallis tests, respectively. Total sample N=3524 (girls + boys). ^2^ 133 missing values; ^3^ 6 missing values; ^4^ BMI for age and sex reference in z score: under/normal weight ≤ +1 SD; overweight/obese: > + 1SD. ^5^ 7 missing values.

Abbreviations: MMW: monthly minimum wages; SD: Standard deviation; IQR: Interquartile range.
